# Searching for Biological Function of the Mysterious PA2504 Protein from *Pseudomonas aeruginosa*

**DOI:** 10.3390/ijms22189833

**Published:** 2021-09-11

**Authors:** Joanna Drabinska, Kamil Steczkiewicz, Martyna Kujawa, Elżbieta Kraszewska

**Affiliations:** Institute of Biochemistry and Biophysics PAS, Pawińskiego 5a, 02-106 Warsaw, Poland; ksteczk@ibb.waw.pl (K.S.); martyna.kujawa@yahoo.pl (M.K.)

**Keywords:** PA2504, TUDOR domain, *Pseudomonas aeruginosa*, sulphur metabolism, crosslink in vivo, RppH, RNA-Seq, Nudix

## Abstract

For nearly half of the proteome of an important pathogen, *Pseudomonas aeruginosa*, the function has not yet been recognised. Here, we characterise one such mysterious protein PA2504, originally isolated by us as a sole partner of the RppH RNA hydrolase involved in transcription regulation of multiple genes. This study aims at elucidating details of PA2504 function and discussing its implications for bacterial biology. We show that PA2504 forms homodimers and is evenly distributed in the cytoplasm of bacterial cells. Molecular modelling identified the presence of a Tudor-like domain in PA2504. Transcriptomic analysis of a *ΔPA2504* mutant showed that 42 transcripts, mainly coding for proteins involved in sulphur metabolism, were affected by the lack of PA2504. In vivo crosslinking of cellular proteins in the exponential and stationary phase of growth revealed several polypeptides that bound to PA2504 exclusively in the stationary phase. Mass spectrometry analysis identified them as the 30S ribosomal protein S4, the translation elongation factor TufA, and the global response regulator GacA. These results indicate that PA2504 may function as a tether for several important cellular factors.

## 1. Introduction

*Pseudomonas aeruginosa* is a Gram-negative bacterium widespread in the environment. Due to its high adaptive abilities, regulated by many poorly characterised mechanisms, it can colonise and infect diverse organisms. This pathogen is commonly present in the hospital environment, where it imposes a great threat to immunocompromised patients, especially those of the chirurgic, intensive care, and neonatal units (reviewed by Spagnolo and colleagues [1]).

Both the cell cycle and virulence of *P. aeruginosa* are dependent on a variety of factors of which secretion systems, quorum sensing, biofilm formation, motility and toxin production have been widely studied (reviewed by Jimenez and colleagues [2]). Despite this, our understanding of these and other processes of *P. aeruginosa* is certainly incomplete. Taking into consideration that almost half of the proteins encoded by its genome still lack annotated function [3], the question arises of which proteins are involved in the processes above-mentioned.

When studying the Nudix type RppH hydrolase of *P. aeruginosa*, we observed that its only partner was the previously uncharacterised protein PA2504.

In *E. coli*, RppH catalyses the removal of pyrophosphate from 5′-triphosphorylated RNA transcripts and via participation in RNA decay regulates expression of different genes [4]. It was later observed that RppH of *P*. *aeruginosa* can functionally substitute for RppH in *E. coli* cells, indicating that, similar to its counterpart, it could also mediate RNA turnover in vivo. The main phenotypic change caused by the absence of RppH in *P. aeruginosa* was a significant increase in the level of the major virulence factor pyocyanin [5].

Up to now, the biological function of PA2504 has not been studied, but it has been observed by different authors that the level of PA2504 transcript changed visibly depending on the conditions of bacterial growth (Table 1).

To elucidate the physiological role of PA2504 in *P. aeruginosa*, we applied a number of techniques including phenotypic analysis of a *PA2504* null mutant and RNA sequencing, in vivo protein crosslinking with mass-spectrometry, and protein structure modelling. We found that the PA2504 homodimer wasevenly distributed in the cytoplasm and most probably serves as an assembly platform for several cellular proteins including TufA (PA4265), S4 protein (PA4239), and GacA (PA2586).

## 2. Results

### 2.1. Phenotypic Characteristic of PA2504 Mutants

Recently, using a *P. aeruginosa* two-hybrid system library [9] and the one-to-one bacterial two-hybrid method (BACTH), we found PA2504 to be the sole partner of the RppH Nudix hydrolase (Figure S3, Supplementary Materials), earlier shown to function as a global regulator influencing many of transcripts including those involved in *P. aeruginosa* virulence [5]. This finding turned our attention to this uncharacterised PA2504 protein.

To establish the influence of PA2504 protein on *P. aeruginosa* functioning, cells carrying chromosomal deletion or overexpressing the PA2504 gene were investigated. To test the effect of the lack of PA2504 protein, the entire PA2504 gene was deleted using pAKE600 suicide vector and overexpression was conducted from an inducible arabinose promoter in the pKGB as described in the Materials and Methods. Single bacterial colonies of each mutant were transferred into L-broth or M9 minimal medium and growth was monitored. No major differences in the growth rate were observed between the mutants and the parental strain in either the exponential or stationary phase. (Figure S2a, Supplementary Materials). 

Furthermore, the lack of PA2504 did not affect bacterial biofilm formation, motility, and the response to any tested antibiotics compared to the parental strain (Figure S2b–d, Supplementary Materials). These results indicate that PA2504 protein had no significant influence on the tested bacterial features.

**Figure 1:**
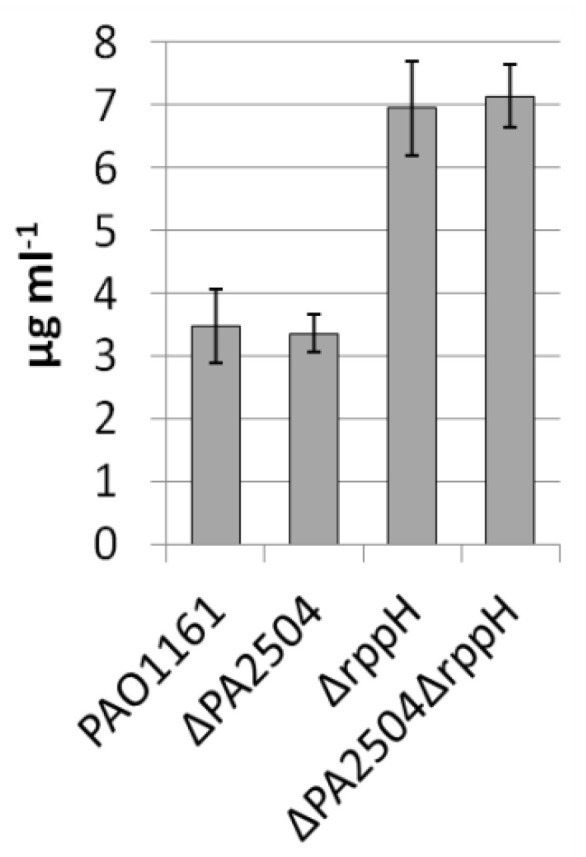
Pyocyanin production by *P. aeruginosa* strains at the stationary phase of growth. Determined as in the “Materials and Methods”. Mean value of at least three independent replicates ±SD is shown.

We have previously shown that RppH influences pyocyanin production in *P. aeruginosa* [5]. To see whether PA2504 is also important in this process, the level of pyocyanin was determined in *ΔPA2504*, *ΔrppH*, and *ΔPA2504ΔrppH* mutants. In *ΔPA2504*, the pyocyanin production was the same as in the parental strain, and in the double mutant, it was similar to that of the single *ΔrppH* mutant, indicating that the lack of PA2504 did not affected the RppH activity in pyocyanin production (Figure 1).

### 2.2. Structural Studies of PA2504 Protein

Since no indications pointing to a possible cellular function of PA2504 appeared from the phenotypic analysis of the mutants, molecular modelling of the protein was performed to search for specific domains in PA2504.

The PA2504 protein contains two domains: N-terminal DUF2314 (PF10077) and C-terminal DUF2185 (PF09951), both found almost exclusively in bacteria (*Firmicutes*, *Proteobacteria*, and many other unclassified bacterial species) and having no known function. According to the Pfam protein families database, the DUF2314-DUF2185 domain tandem is present in multiple *Firmicutes* (*Bacillales*) and *Proteobacteria* (*Alphaproteobacteria*, *Gammaproteobacteria*, and *Burkholderiales*).

**Figure 2:**
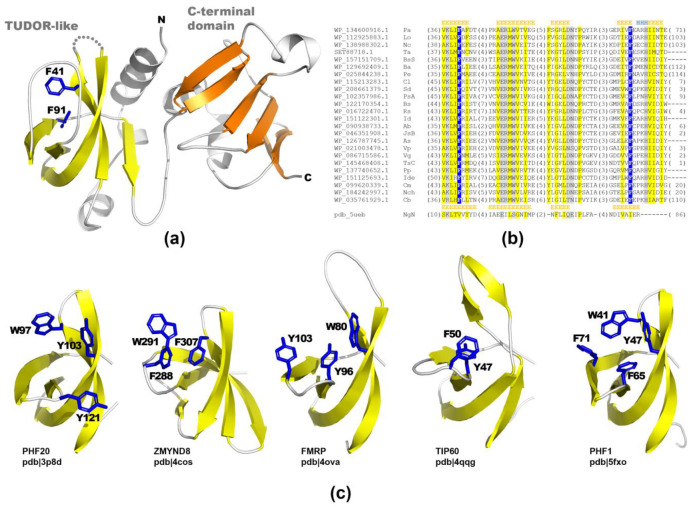
PA2405 contains N-terminal TUDOR-like domain and C-terminal domain of unknown function. (**a**) 3D model of PA2504; β-strands of TUDOR-like and C-terminal domains are in yellow and orange, respectively. Residues forming potential aromatic cage for ligand binding are shownas stick models and coloured blue. (**b**) Multiple sequence to structure alignment of PA2504 homologs and hypothetical protein from *N. gonorrhoeae* of known structure. Residue character conservation marked as follows: uncharged highlighted in yellow, polar in grey, and conserved aromatic residues in blue. Secondary structure predicted for PA2504 and found in the PDB structure are given above the corresponding regions. The number of residues omitted from the alignment is provided in parentheses. (**c**) 3D structures of other TUDOR-like proteins in an orientation corresponding to the PA2504 model in (**a**) (discussed in text).

Hhsearch detected a remote sequence similarity between the N-terminal domain of PA2504 and the TUDOR-like domain of a hypothetical protein from *Neisseria gonorrhoeae* (pdb|5ueb, no publication available) with the score of 34.88 and an estimated probability of 93%. This result was confirmed by the TrRosetta modelling framework, which provided consistent results with a confidence score of 0.71 for the full-length PA2504 (Figure 2a and Figure S4, Supplementary Materials). The C-terminal domain of PA2504 displayed no detectable sequence similarity to any protein of known structure or function. Additionally, the model provided by TrRosetta for this domain was not similar to any protein of known structure, suggesting that it could represent a novel protein fold.

TUDOR domains are widely recognised for their ability to bind modified amino acid residues like methylated lysine within a structure called an aromatic cage—a pocket formed by aromatic residues (Figure 2c). In this manner, they recognise methylated histones (e.g., ZMYND8 (pdb|4cos) [10] and PHF1 (pdb|5xfo) [11]) for gene expression regulation, or bind to other proteins (e.g., PHF20 (pdb|3p8d) [12] binding p53) and protecting it from ubiquitination and, as aconsequence, from degradation or Fragile X mental retardation protein (FMRP) interacting with both tri-methylated lysine and with 82-FIP, one of the FMRP nuclear partners [13,14]. Interestingly, unlike other histone-binding proteins, the model of PA2504 lacked extensive positively charged patches (Figure S5, Supplementary Materials). Overall, it was negatively charged with only a positively charged cleft between the N-terminal TUDOR-like and C-terminal domains (blue area in Figure S5, Supplementary Materials).

### 2.3. Oligomerisation of PA2504

**Figure 3:**
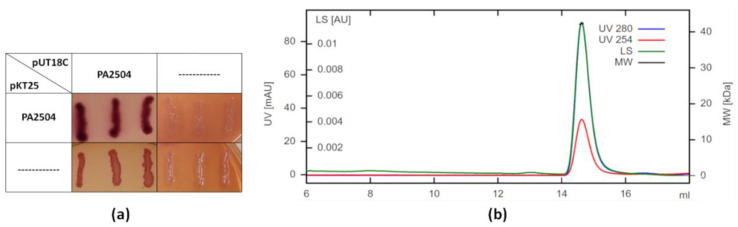
Dimerisation of PA2504 protein. (**a**) Interaction between monomeric forms of PA2504 visualised on MacConkey selective medium (red colonies). (**b**) SEC-MALS analysis of recombinant PA2504 protein. Purified protein was analysed on a Superdex 200 column combined with the light scattering instrument HELEOS as described in the Materials and Methods. LS—light scattering, MW—molecular weight.

To check whether PA2504 is monomeric or forms higher order structures, we investigated its ability to form homo-interactions in vivo using the BACTH system and determined the size of purified PA2504 in solution by size exclusion chromatography combined with multi-angle light scattering (SEC-MALS). Figure 3 shows that PA2504 can oligomerise in vivo and that its SEC-MALS profile corresponds to that of a dimer. In conclusion, it is most likely that PA2504 is also a homodimer in vivo.

### 2.4. Cellular Localisation of PA2504

**Figure 4:**
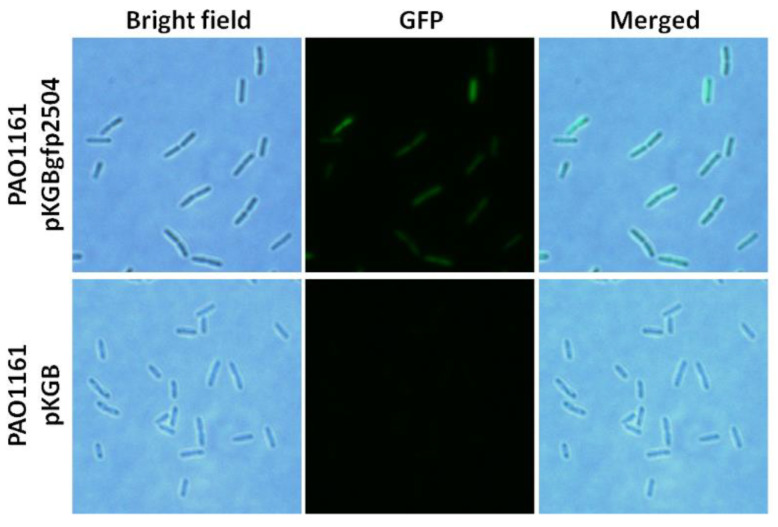
Localisation of GFP-PA2504 protein in *P. aeruginosa* cells. Bright field, GFP fluorescence, and merged are shown. The cells were visualised under a Zeiss Imager. M2 fluorescence microscope as described in theMaterials and Methods.

To localise PA2504 in the cell, GFP tagged PA2504 was expressed in *P. aeruginosa* and observed under a fluorescence microscope. As seen in Figure 4, PA2504 did not associate with any particular cellular structure and was evenly distributed throughout the cells.

### 2.5. Transcriptomic Analysis of ΔPA2504 Mutant

**Figure 5:**
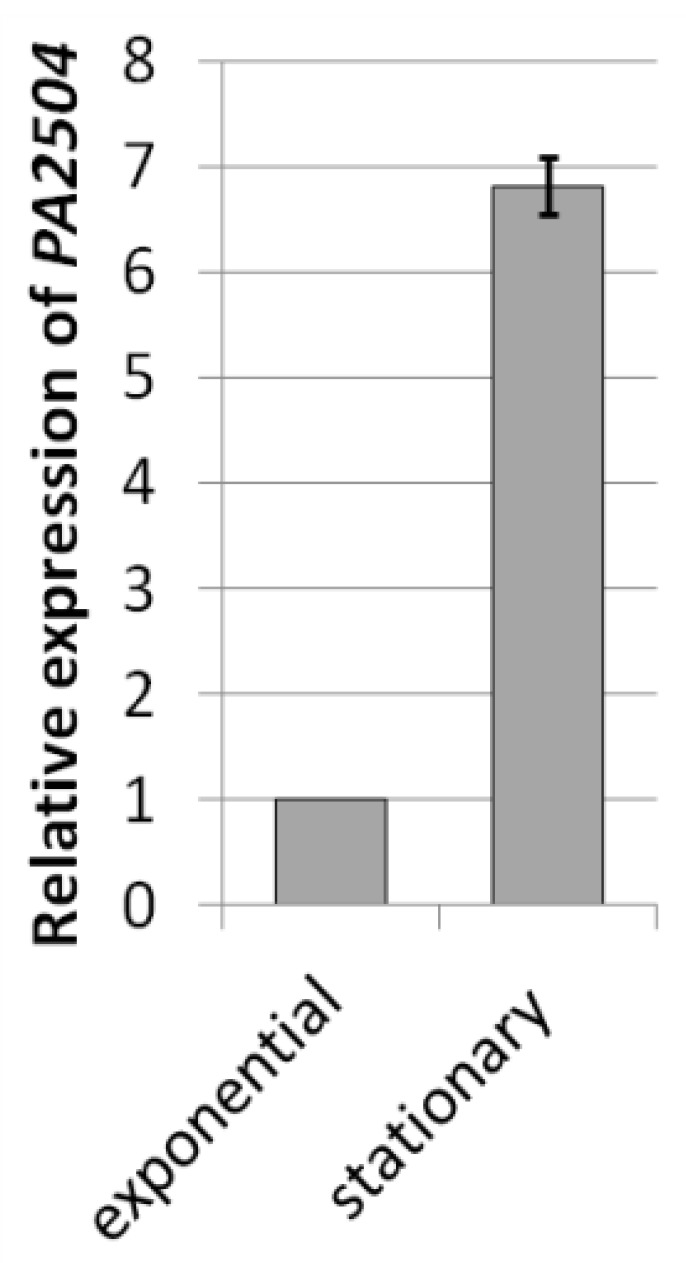
Expression of the *P. aeruginosa PA2504* gene. *PA2504* mRNA was quantified by RT-qPCR in exponential and stationary phase cultures of wild-type *P. aeruginosa* PAO1161 against *nadB* mRNA used as the reference. The relative expression level in the exponential phase was taken as 1. Mean value of three independent replicates ±SE is shown.

Since our preliminary analyses failed to indicate a biological function for PA2504, we compared the transcriptomes of the *ΔPA2504* and the wild-type PAO1161 strains using high-throughput RNA sequencing (RNA-Seq). Since it was found that the level of the *PA2504* transcript was significantly higher (fold change = 6.81) in the stationary phase than in the exponential phase of growth (Figure 5), we compared the transcriptomes in the stationary phase of growth.

**Figure 6:**
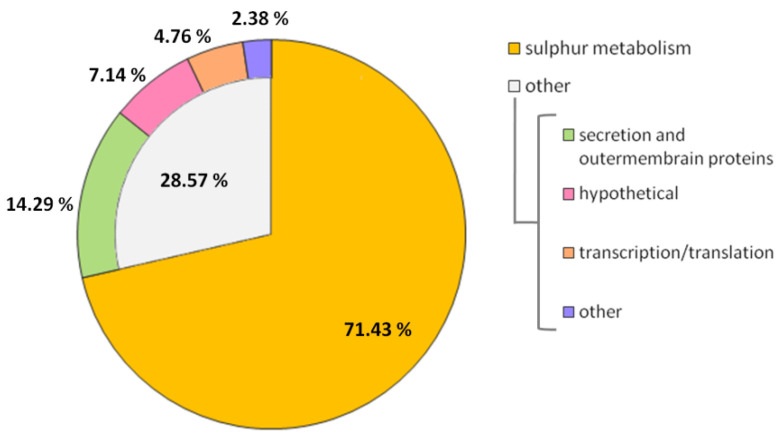
Classification of genes differentially expressed in the *ΔPA2504* mutant compared to wild-type PAO1161. Triplicate samples of each strain were withdrawn from the stationary phase. A total of 42 genes were classified, 30 of them as involved in sulphur metabolism based on the Pseudomonas Genome database or Blast^®^homology.

The lack of the PA2504 protein affected the level of 42 transcripts, of which 41 (97.6%) were downregulated. Notably, 30 differentially expressed genes (71.4%) were related to sulphur assimilation and metabolism (Table 2, Figure 6).

Downregulated transcripts connected to sulphate and thiosulphate import included *cysA*, *cysW*, *cysT*, *sbp*, *cysP*, and *PA2329*; those connected with the transport of aliphatic sulphonic acids were *PA2594* and *PA5103* and with the import of cysteine and methionine *PA2202*, *PA2203*, *PA2204*, *PA3931*, and *PA4195*. The second group related to the conversion of sulphate to sulphite and further to sulphide: *cysD, cysN*, *cysH*, and *cysI*. Furthermore, transcripts coding for enzymes participating in the transformation of L-homocysteine to L-cysteine (review [15]), cystathionine beta-synthase (*PA0399*), and cystathionine gamma-lyase (*PA0400*) were downregulated in the *ΔPA2504* mutant, as was *PA2562*, a homolog of the *E. coli iscS* gene coding for L-cysteine desulphurase [16] (Table 2).

### 2.6. Growth of ΔPA2504, ΔrppH, and ΔPA2504ΔrppH Mutants on Different Sulphur Sources

**Figure 7:**
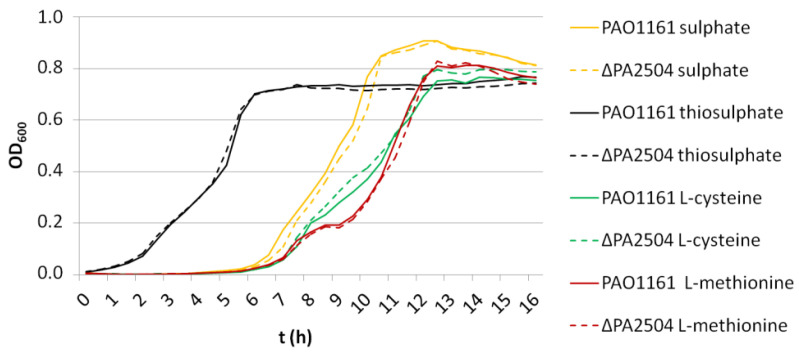
Growth of *P. aeruginosa* strains on *ΔPA2504* mutant in modified M9 minimal medium supplemented with different sulphur sources (0.5 mM).

The transcriptomic analysis suggested that PA2504 could be involved in sulphur transport and metabolism. Therefore, we compared the growth of the *ΔPA2504* and PAO1161 parental strains on M9 minimal medium supplemented with different sulphur source such as sulphate, thiosulphate, or amino acids cysteine and methionine. Surprisingly, no major differences were seen between the two strains in the exponential or stationary phase of growth regardless of the sulphur source (Figure 7).

To confirm these results, the Biolog system, which allows a simultaneous measurement of bacterial growth on many different sulphur sources, was used. Again, no significant differences in growth between the mutant and the parental strain were observed (Figure S6, Supplementary Materials).

Since PA2504 is the partner of RppH, whose involvement in sulphur metabolism was noticed [17], we asked whether the absence of RppH could affect growth on various sulphur sources in the presence or absence of PA2504. The growth curves of *ΔPA2504*, *ΔrppH*, and the double mutant *ΔPA2504ΔrppH* on different sulphur sources were determined. Notably, the *ΔrppH* strain showed slower growth on cysteine, methionine, taurine, and MOPS than *ΔPA2504* and the parental strain, but this effect was not influenced by the absence of PA2504 (strain *ΔPA2504ΔrppH*) (Figure S7, Supplementary Materials).

Taken together, these results show that despite affecting the expression of numerous sulphur related genes, PA2504 is not involved in the transport or metabolism of any of the tested sulphur compounds.

### 2.7. Search for PA2504 Cellular Partners

To further search for PA2504 function, we attempted to identify its protein partners other than RppH. To this end, His-tagged *PA2504* was expressed in *P. aeruginosa* grown to exponential or stationary phase, in vivo protein crosslinking was performed, and PA2504 proteins were separated by electrophoresis following crosslink reversal as described in the Materials and Methods.

**Figure 8:**
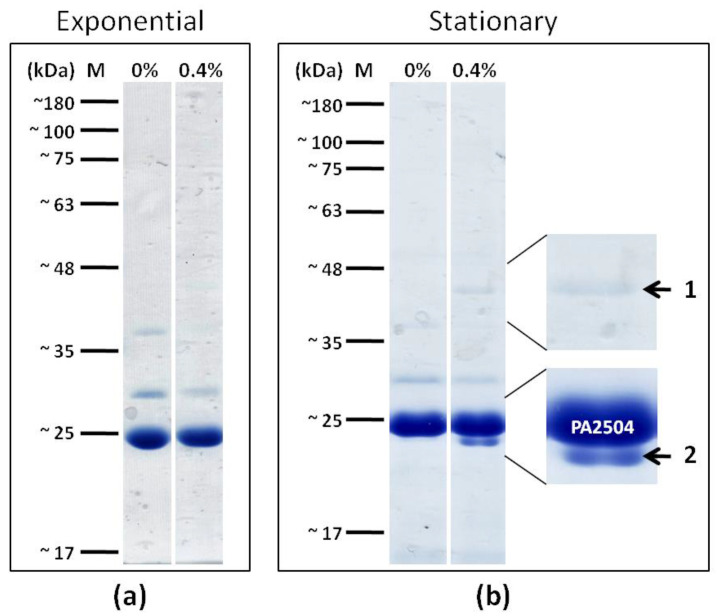
Search for PA2504 partners. Expression of PA2504 was induced with 0.2% arabinose in *P. aeruginosa ΔPA2504* carrying pKGB2504. The potential PA2504 complexes were crosslinked with 0.4% formaldehyde for 10 min, and purified as described in the Materials and Methods. Crosslinks were reversed by heating at 100 °C for 10 min and the proteins were separated by SDS-PAGE. (**a**) Proteins interacting with PA2504 in the exponential phase. (**b**) Proteins interacting with PA2504 in the stationary phase. In the enlarged fragments, arrows indicate additional bands specific to stationary phase located between 35 kDa and 45 kDa and below 25 kDa. Uncropped photos of the gels are presented in Supplementary Figure S8.

Interestingly, following crosslink, two protein bands appeared specifically in the stationary phase of culture growth (Figure 8).

A mass spectrometry analysis of the first band identified, with high confidence, based on the mascot score and emPAI values, the 43.4 kDaTufA translation elongation factor (PA4265), while in the second band, the presence of the 23.3 kDa S4 ribosomal protein (PA4239) and the 23.6 kDa GacA transcription regulator were detected. The results of mass spectrometry analysis are presented in Table S3 in the Supplementary Materials.

Interestingly, RppH was not observed under the experimental conditions used, suggesting that if the interaction between RppH and PA2504 occurs in *P. aeruginosa* cells, it happens under different circumstances.

## 3. Discussion

A variety of techniques were used to search for the biological function of PA2504 protein, the alleged only partner of the RppH hydrolase from *P. aeruginosa*. A lack or over-production of PA2504 did not affect bacterial growth in various experimental conditions, nor was there an influence of PA2504 on biofilm formation, motility, or antibiotic resistance. In contrast to RppH, whose absence dysregulates pyocyanin production [5], a lack of PA2504 did not affect it, suggesting that pyocyanin synthesis does not require the presence of the putative PA2504/RppH protein complex. Additionally, PA2504 did not associate with any particular cellular structure that could point into its function.

In addition, despite the results of a transcriptomic analysis strongly suggesting an indirect involvement of PA2504 in sulphur metabolism, its absence alone or in combination with RppH did not affect bacterial growth on a large array of sulphur compounds. However, taking into consideration the substantial number of sulphur derivatives found in nature and the enormous environmental adaptability of *P. aeruginosa*, one cannot exclude that PA2504 does in fact participate in a yet unrecognised sulphur pathway. In this context, our finding that the absence of RppH hampered growth on some of the sulphur sources tested seems interesting and worthy of further studies.

Having found virtually no physiological consequences of PA2504 absence, we were nevertheless able to gain some insight into its possible role by identifying its in vivo protein partners. Notably, these interactions appeared to be specific to the stationary phase of growth, when PA2504 is known to be upregulated. The partners included the elongation factor TufA (EF-TuA), the ribosomal protein S4, and the global response regulator GacA.

S4 ribosomal protein is essential for 30S ribosome assembly (for review see [18]). Mutation of the gene encoding this protein increases the level of translation errors [19]. Apart from its role in ribosome biogenesis, S4 may function as a general anti-termination factor in transcription [20].

Similarly to S4, the elongation factor EF-Tu, whose canonical role is to transport aminoacylated tRNA to the ribosome [21], has evolved the ability to perform other functions. Its involvement in cell adhesion and biofilm formation, pathogenesis, and stringent response has beenreported [22,23,24] and possibly the list of diversities is not close yet.

GacA is a component of the global signal transduction system GacS/GacA highly conserved in Gram-negative bacteria. This regulatory system is required for the production of many secondary metabolites and extracellular enzymes including virulence factors and biocontrol factors linked with the adaptability to the environment [25]. In addition, a transcriptomic analysis of a *P. aeruginosa gacA* mutant also showed that transcripts coding for proteins of primary metabolism including those involved in sulphur metabolism were affected [26].

Interestingly, as shown by molecular modelling, PA2504 contains a Tudor-like domain. Tudor domain proteins, identified and extensively studied in eukaryotes, function as molecular adaptors, binding methylated arginine or lysine residues on their substrates to promote physical interactions and assembly of macromolecular complexes participating in diverse cellular pathways mostly connected with nucleic acid metabolism. Moreover, it was observed that the specificity of some Tudor domain proteins depend on their ability to form homodimers (review, [12,27]). Although similar complexes are yet to be found in prokaryotic cells, Tudor-like domains have been identified in several bacterial species [28,29,30,31]. There is also increasing evidence for protein arginine methylation in prokaryotes. Recently, a proteomic analysis found methylated arginine in the outer membrane protein TamA of *E. coli* [32] and *Mycobacterium tuberculosis* methylation at lysine and/or arginine residues was identified in nine proteins including MtrA, an essential response regulator of a two-component signalling system. The methylation of MtrA attenuated its binding to DNA [33]. In addition, it was shown that trimethylation of lysine 5 of EF-Tu was important for initial adhesion of *P. aeruginosa* cells to host epithelium [34]. Moreover, it was noticed that this modification had no impact on the primary function of EF-Tu [35], suggesting that depending on posttranslational modifications, the protein may play a different role.

Although, the Tudor-like domain of PA2504 seems to retain an aromatic cage, probably for accepting modified amino acids of other proteins, its more detailed biological function remains elusive. Proteins interacting with histones present extensive positively charged patches on their surfaces [10,11] and those specialised in binding to one well-defined protein display unique electrostaticpatterns [12,13,14]. It appears that PA2504 is negatively charged at its surface, which suggests that it will rather not interact with proteins immediately attached to nucleic acids. On the other hand, it might hijack positively charged nucleic acids-binding proteins and block their native functions.

Further studies are needed to explain in detail how homodimeric PA2504 influences the biological function of S4, EF-TuA, and GacA, but it could be speculated that it might be required to bind these factors in order to fine tune cellular response to external conditions (e.g., stationary phase-dependent nutrient shortage).

## 4. Materials and Methods

### 4.1. Bacterial Strains and Growth Conditions

The *E. coli* and *P. aeruginosa* strains used in this study are listed in Table S1 in the Supplementary Materials and the plasmids in Table 3. Bacteria were grown routinely in Luria-Bertani (L-broth) medium or on L-agar (L-broth with 1.5% (wt/vol) agar) at 37 °C. To determine growth on different sulphur sources, *P. aeruginosa* strains were grown in modified M9 minimal medium (33.7 mM Na_2_HPO_4_, 22 mM KH_2_PO_4_, 8.55 mM NaCl, 9.35 mM NH_4_Cl, 1.0 mM MgCl_2_, 0.3 mM CaCl_2_, 152 mM leucine, 134 µM FeCl_3_, 20 mM sodium citrate), and supplemented with appropriate sulphur sources (0.5 mM). Growth curves were obtained with the use of a Varioscan Lux multimode plate reader (Thermo Scientific™) in 96-well plates.

Where needed, appropriate antibiotics were added to the media as follows: ampicillin, 100 µg mL^−1^ for Ap^R^ in *E. coli*, kanamycin sulphate, 50 µg mL^−1^ for Km^R^ in *E. coli*, 25 µg mL^−1^ chloramphenicol for Cm^R^ in *E. coli*, and 200 µg mL^−1^ in *P. aeruginosa*; carbenicillin disodium salt, 300 µg mL^−1^ for Cb^R^ in *P. aeruginosa*; rifampicin, 300 µg mL^−1^ for Rif^R^ in *P. aeruginosa*.

### 4.2. Deletion of PA2504

*ΔPA2504* and *ΔPA2504ΔrppH* mutants were obtained as follows: *PA2504* upstream and downstream DNA fragments of about 300–500 nucleotides each were amplified by PCR using chromosomal DNA as a template and subsequently ligated into the suicide pAKE600 vector. pAKE600 carries the pMBIori, allowing replication in *P. aeruginosa* [37]. *E. coli* S17-1 was transformed with the obtained pAKE2504 plasmid and the transformants were conjugated with *P. aeruginosa* PAO1161 (for *ΔPA2504*) or *ΔrppH* (for *ΔPA2504ΔrppH*) using the procedure described by [40]. Following removal of the integrated suicide vector, *P. aeruginosa* colonies were analysed by RT-PCR to determine whether the allele exchange was successful, and the transcript of the gene was absent (Figure S1, Supplementary Materials).

### 4.3. Overproduction of PA2504

The *PA2504* gene without the start codon was cloned into the pQE-80L vector to obtain a His_6_PA2504 fusion. The *His_6_PA2504* fragment was cloned into the pKGB vector under the control of an arabinose inducible promoter to obtain the pKGB2504 plasmid. The obtained plasmid was introduced into suitable *P. aeruginosa* strains by conjugation as stated above. For protein overproduction, overnight cultures of *P. aeruginosa* carrying the plasmid were diluted in L-broth or M9 medium 1:100 and protein production was induced by the addition of 0.02% arabinose.

### 4.4. Pyocyanin Quantification

Overnight cultures of *P. aeruginosa* PAO1161 and mutant strains were inoculated 1:100 in 20 mL of L-broth and grown in triplicate at 37 °C with aeration. After 12 h of incubation, two 7.5 mL samples were withdrawn from each culture and extracted with 4.5 mL of chloroform and then 1.5 mL 0.2 M HCl was added to the extract, causing the colour change. OD_520_ was determined and the obtained values were converted to pyocyanin content following [41]. The experiment was repeated at least three times.

### 4.5. Molecular Protein Modelling

Sequence similarity searches were performed using hhsearch, a highly sensitive meta profile comparison engine for remote homology detection [42]. Additionally, the full-length PA2504 protein was modelled with TrRosetta [43], which combines energy minimisation with restraints estimated by the neural network. Multiple sequence alignments were obtained with Mafftlinsi flavour [44] for accuracy. Secondary structure was predicted using PSIPRED [45]. Proteins similar in structure to the PA2504 model were identified using the DALI server [46]. Electrostatic analysis was done with the APBS [47] plugin to PyMOL. All 3D structure visualisations were prepared in PyMOL.

### 4.6. Purification of His_6_-Tagged PA2504 by Affinity Chromatography

The pQE2504 plasmid carrying the *PA2504* gene coding a protein in fusion with a His_6_-tag was introduced into *E. coli* BL21-DE3. An overnight culture of *E. coli* transformant was diluted 1:50 in 100 mL of L-broth and grown at 37 °C to OD_600_ = 0.6. Then, IPTG was added to 0.2 mM, cells were grown for the next 3–4 h, and pelleted by centrifugation for 10 min at 4 °C. The pellets were suspended in 8 mL of sonication buffer (300 mM NaCl, 100 mM Tris-Cl, pH 7.5, 5 mM β-mercaptoethanol) containing protease inhibitors (Roche, Basel, Switzerland or Sigma Aldrich Saint Louis, MO, USA) and disrupted by sonication (5 × 1 min). The obtained cell extract was cleared by centrifugation at 4 °C for 30 min at 14,000 *g*. The supernatant was incubated with 200 µL of Ni^2+^-NTA resin (Sigma Aldrich, Saint Louis, MO, USA) for 2 h with gentle shaking at 4 °C. The resin was washed twice with sonication buffer, then three times with sonication buffer containing 10 mM imidazole (Sigma Aldrich, Saint Louis, MO, USA), and three times with sonication buffer containing 20 mM imidazole. Resin-bound proteins were then eluted with three 200 µL portions of 250 mM imidazole and dialysed overnight against dialysis buffer (50 mM Tris-Cl, pH 7.0, 2 mM EDTA, 2 mM DTT). Protein purity was analysed by SDS-PAGE and concentration measured using the Bradford reagent [48].

### 4.7. SEC-MALS Analysis

The purified recombinant PA2504 protein was loaded onto a Superdex200 column combined with a multi-angle light scattering instrument HELEOS (WYATT Technology, Santa Barbara, CA, USA). The proteins were eluted with 50 mM Tris-Cl buffer, pH 7.0, 300 mM NaCl, at a flow rate of 0. 5 mL min^−1^. Each fraction was automatically analysed by multi-angle light scattering.

### 4.8. Bacterial Two-Hybrid System (BACTH)

DNA fragments encoding PA2504 and RppH were PCR-amplified using *P. aeruginosa* genomic DNA as template with appropriate primers (Table S2). The obtained fragments were cloned into the plasmids of the BACTH system [38]. Resulting plasmids pKT2504, pNTrppH, and p18C2504 were co-transformed into the *E. coli* BTH101 *cyaA* strain as needed. The transformants were analysed on MacConkey selective medium plates with maltose. As negative controls, the BTH101 strain transformed with one empty vector and one encoding the hybrid protein was used.

### 4.9. Bacterial Two-Hybrid Library Screening

A library of the *P. aeruginosa* PAO1161 genome in the pUT18C plasmid was used [9]. Briefly, DNA from *P. aeruginosa* PAO1161 was fragmented with helium at 0.9 Pa for 4 min, precipitated, dried, and dissolved in water. The DNA fragments were treated with Klenow DNA polymerase I and T4 phage polymerase and then ligated into the SmaI-treated and dephosphorilated pUT18C BACTH vector. The obtained plasmids were introduced into the *E. coli* cells. The bacteria were collected to obtain a 10-fold coverage of the whole *P. aeruginosa* PAO1161 genome and inoculated into LB medium for library isolation. Three milligrams of plasmid DNA library was used for further experiments.

The pNTrppH plasmid and the pUT18C library were co-transformed into the *E. coli* BTH101 *cyaA* strain. A four-step verification was used. First, the transformed cells were spread on LB plates with 0.5 mM IPTG and 40 µg mL^−1^ X-Gal. The plates were incubated for five days at 28 °C. Blue colonies were replicated on MacConkey selection medium and incubated 48 h in 28 °C. Plasmids where isolated from streaks thatchanged in colour to red. The obtained plasmids were co-transformed with pNTrppH into *E. coli* BTH101 *cyaA* and the bacteria were spread on LB X-Gal IPTG plates. Library plasmids from blue colonies were sequenced in house and the sequences were verified for protein-coding correct frame orientation. When the above conditions were met, the protein encoded by the fragment was considered as a potential partner of RppH. To confirm the interactions, the entire genes encoding the identified proteins were cloned in pUT18C and then co-transformed with pNTrppH into the *E. coli* BTH101 *cyaA* and selected LB X-Gal IPTG medium. In each step of the procedure a positive and a negative control was used.

For the identification of the protein partners of RppH, 21 co-transformations of the pNTrppH with the pUT18C library were performed. Of the approximately 35,000 colonies analysed, 24 showed a change incolour. After MacConkey medium selection, eight clones were selected for further analysis. Upon re-analysis, only two clones showed a change incolour on the selection medium and upon sequence analysis, only one carrying the *PA2504* gene encoding an unknown protein was accepted. The interaction of PA2504 with RppH was confirmed as detailed above.

### 4.10. Cellular Localisation of PA2504

PA2540 protein was localised in *P. aeruginosa* cells by cloning the *PA2504* and *sfGFP* (super folder GFP) fragments in to the pKGB to give pKGBgfp2504. *PA2504* was amplified on the PAO1161 strain genomic DNA template and *sfGFP* fragment on the pBAD24-sfGFPx1 plasmid [39]; pKGBgfp2504 was introduced into the *P. aeruginosa ΔPA2504* by conjugation [40]. An overnight culture was diluted 1:150 and incubated for 4 h at 37 °C, then 1.5 mL of the culture was centrifuged and resuspended in 20 µL of fresh LB medium and 1 µL of the suspension was placed on a microscope slide covered with polylysine (Thermo Scientific). Cells were studied using a Zeiss Imager. M2 fluorescence microscope with a 100× 1.30 NA Plan-Neofluar lens and Zeiss AxioCam MRc5 camera with ta 470/40 nm excitation filter, 495 nm dichroic beam-splitter, and 525/50 nm emission filter. AxioVision (AxioVs40 V 4.8.2.0, Carl Zeiss MicroImaging) software was used.

### 4.11. In Vivo Protein Crosslinking and Purification of Protein Complexes

Crosslinking experiments were based on [49]. The minimal concentration of formaldehyde and incubation time required to induce sufficient crosslinking were determined experimentally. *P. aeruginosa ΔPA2504* strain carrying the pKGB2504 plasmid, encoding His_6_-taged PA2504, was used. An overnight culture was diluted 1:100 in fresh L-broth medium with 0.2% arabinose and chloramphenicol and grown for 18 h at 37 °C with vigorous shaking. Formaldehyde was added to cultures in a range of concentrations from 0.1% to 1% and the bacteria were incubated for 15 min at room temperature with gentle shaking every 3 min. The formaldehyde was quenched by the addition of 1/10 culture volume of ice cold 0.125 M glycine in PBS and 100 mL of each culture was centrifuged in 5000 rpm for 30 min, washed with 50 mL of cold PBS, and centrifuged again. The pellets were suspended in 8 mL of sonication buffer (300 mM NaCl, 100 mM Tris-Cl, pH 7.5, 5 mM β-mercaptoethanol) containing protease inhibitors (Sigma Aldrich, Saint Louis, MO, USA) and disrupted by sonication (5 × 1 min). The obtained cell extracts were cleared by centrifugation at 4 °C for 30 min at 14,000 *g*. Supernatants were incubated with 200 µL of Ni^2+^-NTA resin (Sigma Aldrich, Saint Louis, MO, USA) for 2 h with gentle shaking at 4 °C. The resin was washed twice with sonication buffer and then three times with sonication buffer containing 10 mM imidazole (Sigma Aldrich, Saint Louis, MO, USA) and three times with sonication buffer containing 20 mM imidazole. His_6_-tagged PA2504 crosslinked with its protein partners was eluted from the nickel resin with three portions of 200 µL of 400 mM imidazole (then pooled). The proteins were incubated with loading dye at 65 °C for 20 min, which preserves the crosslinking [50]. The crosslinked proteins were separated on SDS-PAGE gel followed by western blotting. Aconcentration of 0.4% formaldehyde and 10 min incubation were chosen for further experiments based on the visibility of the PA2504 dimer and larger complexes. For mass spectrometry identification of the crosslinked proteins, the Ni–NTA-isolated complexes were incubated with the loading dye for 10 min at 100 °C, which disrupted the formaldehyde crosslinks. Proteins from three separate biological replicates treated with 0.4% formaldehyde and three not treated with formaldehyde were separated in 12% SDS-PAGE gel.

### 4.12. Mass Spectrometry

The bands of interest were cut out from the gel and fragmented to 1–2 mm pieces with sterilised scalpel. For Coomassie Brilliant blue removal, gel fragments were covered with destaining solution (50% acetonitrile in 50 mM NH_4_HCO_3_) and vortexed until fully destained. The gel fragments were dried with 100% acetonitrile, followed by cysteine reduction with a solution of 10 mM DTT, 100 mM NH_4_HCO_3_ for 30 min in 57 °C. The gel was dried again as previously described and cysteine alkylation was performed by 45 min of incubation in alkylation solution (50 mM iodoacetamid, 100 mM NH_4_HCO_3_). Any residue of used solutions were washed away with 100 mM NH_4_HCO_3_ and subsequently with 100% acetonitrile, used twice, alternately. The gel was dried again as previously described. The dry gel fragments were covered with a trypsin solution (10 ng/µL in 25 mM NH_4_HCO_3_) and incubated at 37 °C overnight. The obtained peptides were extracted with 30 µL of 0.1% trifluoroacetic acid, and 0.2% acetonitrile solution. The peptide mixture was separated with liquid chromatography, followed by mass measurements with an Orbitrap spectrometer (Thermo). The peptides were annotated to the *P. aeruginosa* proteome with the use of the *Pseudomonas* genome data base [3] with the use of MASCOT (http://www.matrixscience.com (accessed on 22 April 2021 and 9 August 2021)).

### 4.13. RNA Isolation

For isolation of total cellular RNA for next-generation sequencing (RNA-Seq) or RT-qPCR, *P. aeruginosa* PAO1161 and *ΔPA2504* strains were inoculated 1:100 in fresh L-broth and incubated for 18 h with shaking at 37 °C, then 1.5 mL samples were taken from three independent biological replicates and immediately treated with RNA protect Bacteria Reagent (Qiagen, Hilden, Germany) and spun down. RNA was isolated from the cell pellet with the RNeasy Mini Kit (Qiagen) and digested with DNase I using the RapidOut DNA Removal Kit (Thermo Scientific, Waltham, MA, USA). RNA quality and integrity was assessed with a bioanalyzer (Agilent Technology, Santa Clara, CA, USA), and concentration was estimated using a Nano Drop ND-1000 spectrophotometer.

### 4.14. RT-qPCR

Total RNA (800 ng) from three biological replicates of each strain was used for cDNA synthesis using a QuantiTect Reverse Transcription Kit (Qiagen). The cDNA then served as a template for qPCR with gene-specific primers (Table S2, Supplementary Materials) and 5× HOT FIREPolEvaGreen qPCR Mix Plus (Solis Biodyne) in a LightCycler 480 II System (Roche Molecular Diagnostics). Relative transcript level was determined by a comparisonof crossing points (Cp) for the target and the reference gene (*nadB*). Three technical repetitions were undertakenfor each primer pair. The ratio/fold change was calculated using Pfaffl’s formula [51].

### 4.15. RNA-Seq Analysis

RNA as above prepared was subjected to next-generation sequencing by a commercial provider. Ribosomal RNA was depleted using QIAseqFastSelect (Qiagen). cDNA libraries were prepared with the NEBNext^®^ Ultra ™ II Directional RNA Library Prep Kit for Illumina^®^ (New England Biolabs) with information about the transcription direction preserved and sequenced on a NextSeq500 device (Illumina) with 75-nt paired-end reads.

### 4.16. Bioinformatic Analysis of RNA-Seq Results

First, for each file with raw sequencing data, a data quality report was prepared with the use of FASTQC [52]. The reads were mapped with TopHat program [53] to the *P. aeruginosa* PAO1 genome using the fr-firststrand option and in the nonovel-juncs mode. The percentage of reference mapping reads was then verified. The number of mapped base pair reads for individual genes was counted with HTseq [54] with distinction considering the transcript strand (–stranded=reverse). Genes were annotated based on the *P. aeruginosa* PAO1 gene descriptions from PseudoCap. Final results were prepared in the R environment (https://www.r-project.org/ (accessed on 9 June 2020)) with the use of the DESeq2 package [55]. Differential expression was analysed statistically with the Walds test. Obtained *p*-values were FDR-adjusted using the Benjamini–Hochberg method [56]. The RNA-Seq results were deposited at NCBI’s Gene Expression Omnibus [57] and are accessible via the GEO Series Accession Number GSE179150 at [58] (https://www.ncbi.nlm.nih.gov/geo/query/acc.cgi?acc=GSE179150).

## Figures and Tables

**Table 1:** Cellular level of PA2504 transcript in different conditions of bacterial growth. Listed are the conditions from which the samples were withdrawn, the *P. aeruginosa* strains used for the experiment (Strain), fold change of *PA2504* gene expression (FC), and source of the data.

Conditions	Strain	FC	Source
Burn wound isolate (human) vs. stationary growth in rich liquid medium	clinical isolate	−11.0	[6]
Cystic fibrosis patient lung isolate vs. stationary growth in rich liquid medium	clinical isolate	−9.0	[6]
*ΔvqsR* vs. WT, ABC minimal medium	PAO1	+8.0	[7]
Murine tumour isolate (mouse) vs. stationary growth phase in rich liquid medium	veterinary isolate	−6.3	[6]
GUN (glucose uptake null) + glucose vs. WT	PAO1	+3.6	[8]
GUN (glucose uptake null) + glucose vs. WT + glucose	PAO1	+2.8	[8]

**Table 2:** Transcripts differentially expressed in *ΔPA2504*.

Gene ID	Name	Log_2_ FC	Product Description
**PA0280**	*cysA*	−2.70	Sulphatethiosulphate ABC transporter ATP-binding protein CysA
**PA0281**	*cysW*	−2.76	Sulphate transporter CysW
**PA0282**	*cysT*	−2.79	Sulphate transporter CysT
**PA0283**	*sbp*	−3.13	Sulphate-binding protein
**PA0284**	*oscA*	−3.11	Hypothetical protein/*sulphur starvation response protein*
**PA0399**	*PA0399*	−0.73	Cystathionine beta-synthase
**PA0400**	*PA0400*	−0.54	Cystathionine gamma-lyase
PA1245	*aprX*	−0.92	Extracellular protease AprX
PA1246	*aprD*	−0.70	Alkaline protease secretion ATP-binding protein AprD
PA1247	*aprE*	−0.64	Alkaline protease secretion protein AprE
PA1248	*aprF*	−0.73	Alkaline protease secretion protein AprF
PA1249	*aprA*	−0.83	Alkaline metalloproteinase
**PA1493**	*cysP*	−0.55	Sulphate ABC transporter substrate-binding protein
**PA1756**	*cysH*	−0.98	Phosphoadenosinephosphosulphate reductase
**PA1837**	*PA1837*	−1.52	Hypothetical protein/*oxidoreductase probably involved in sulphite reduction*
**PA1838**	*cysI*	−1.29	Sulphite reductase
PA1912	*femI*	−0.39	ECF sigma factor FemI
**PA2062**	*PA2062*	−1.00	Probable pyridoxal-phosphate dependent protein/*IscS subfamily cysteine desulphurase*
PA2086	*PA2086*	−1.12	Epoxide hydrolase
**PA2202**	*PA2202*	−1.79	Amino acid permease
**PA2203**	*PA2203*	−2.32	Amino acid permease
**PA2204**	*PA2204*	−3.02	ABC transporter
PA2328	*PA2328*	−0.86	Hypothetical protein/*nitrate transport protein NrtA precursor*
**PA2329**	*PA2329*	−0.72	ABC transporter ATP-binding protein/*nitrate/sulphonate/bicarbonate ABC transporter ATPase*
**PA2330**	*PA2330*	−0.74	Hypothetical protein/*acyl-CoA/acyl-ACP dehydrogenase*
**PA2426**	*pvdS*	−0.43	Extracytoplasmic-function sigma-70 factor
**PA2481**	*PA2481*	+0.46	Hypothetical protein/*thiosulphate dehydrogenase*
**PA2594**	*PA2594*	−1.40	Putative periplasmic aliphatic sulphonate binding protein
**PA2598**	*PA2598*	−0.70	Hypothetical protein/*methanesulphonate monooxygenase*
PA2786	*PA2786*	−1.10	Hypothetical protein/*GAF domain-containing protein*
**PA3441**	*ssuF*	−3.07	Molybdopterin-binding protein/*organosulphonate utilisation protein SsuF*
**PA3931**	*PA3931*	−2.58	Putative methionine-binding protein
PA3932	*PA3932*	−0.97	Transcriptional regulator
PA4067	*oprG*	−0.49	Outer membrane protein OprG
**PA4195**	*PA4195*	−1.64	Putative amino acid ABC transporter substrate-binding protein/*ABC transporter glutamine-binding protein GlnH precursor*
**PA4442**	*cysN*	−1.87	Bifunctional sulphate adenylyltransferase subunit 1/adenylylsulphate kinase
**PA4443**	*cysD*	−2.22	Sulphate adenylyltransferase subunit 2
**PA4470**	*fumC1*	−0.29	Fumarate hydratase
PA4471	*PA4471*	−0.58	Hypothetical protein
**PA5024**	*ytnM*	−0.44	Hypothetical protein/*sulphite exporter TauE/SafE*
**PA5025**	*metY*	−0.62	*O*-acetylhomoserineaminocarboxypropyltransferase
**PA5103**	*puuR*	−1.03	Hypothetical protein/*PhnD/SsuA/transferrin family substrate-binding protein*

Listed are transcripts with the log_2_ fold change (FC) (differences statistically significant; with FDR-adjusted *p* ≤ 0.05). In bold—genes related to sulphur metabolism. In italics are descriptions of close homologs of *P. aeruginosa* genes identified using the BLASTP^®^ program.

**Table 3:** Plasmids used in this study.

Plasmid	Relevant Features	Source
pKGB	pKGB8.0.2 vector, in this work referred to as pKGB; *araBADp, araC*, Cm^R^, broad-host-range expression vector	[36]
pQE-80L	*ori_ColE1_*ApR T5p *lacOlacIq* His6 tag, expression vector	Qiagen
pAKE600	*ori_MB1_ ori_RK2_*Ap^R^*sacB*	[37]
pKT25	*ori P_15A_,* Km^R^*, lacp–cyaT25*	[38]
pKNT25	*orip15, Km^R^, lacp–cyaT25*	[38]
pUT18C	*ori_ColE1_,* Ap^R^*, lacp–cyaT18*	[38]
pBAD24-sfGFPx1	*araBADp*, *araC*, Ap^R^, Superfolder GFP ORF cloned into pBAD24 for expression in *E. coli*	[39]
pAKE2504	pAKE600 plasmid with 275 bp upstream region of *PA2504* gene with start codon and 253 bp downstream region of *PA2504* gene with stop codon, inserted as EcoI-PstI and PstI-BamHI fragments	This work
pQE2504	pQE-80L with *PA2504* without start codon, inserted as BamHI-SalI fragment	This work
pKGB2504	pKGB with *PA2504*Hisx6 inserted as EcoRI-SalI fragment	This work
pKGBgfp2504	pKGB with *sfGFP* without stop codon, inserted as an EcoRI-HindIII fragment, and *PA2504* without start codon, cloned as XbaI-SacI fragment	This work
pKT2504	pKT25 with *PA2504* without start codon, inserted as BamHI-EcoRI fragment	This work
pNTrppH	pKNT25 with *PA2504* without stop codon, inserted as BamHI-EcoRI fragment	This work
p18C2504	pUT18C with *PA2504* without start codon, inserted as BamHI-EcoRI fragment	This work

## Data Availability

The RNA-Seq results were deposited in NCBI’s Gene Expression Omnibus and are accessible through GEO Series accession number GSE179150 (https://www.ncbi.nlm.nih.gov/geo/query/acc.cgi?acc=GSE179150 (accessed on 1 July 2021)).

## Supplementary Materials

The following are available online at https://www.mdpi.com/article/10.3390/ijms22189833/s1. All supplementary materials are provided in a single pdf file. The content is as follows: Supporting Materials and Methods; Table S1: Bacterial strains used in this study; Table S2: Primers used in this study; Table S3: Proteins identified by mass spectrometry analysis of bands cut from SDS-PAGE gel; Figure S1: Control of *PA2504* gene deletion; Figure S2: Effect of PA2504 depravation and overproduction on growth, biofilm production, motility, and antibiotic susceptibility; Figure S3: Interaction between PA2504 and RppH; Figure S4; Figure S5: Molecular surfaces of PHF1, ZMYND8, and PA2504 coloured according to calculated electrostatic potential; Figure S6: Effect of PA2504 depravation and overproduction in the presence of different sulphur sources; Figure S7: Growth curves of the *P. aeruginosa* wild-type PAO1161 strain and *ΔrppH*, *ΔPA2504*, *ΔPA2504ΔrppH* mutantstrains in M9 minimal medium supplemented with a sulphur source; Figure S8: Uncropped pictures of SDS-PAGE gels used to prepare Figure 8.

## Abbreviations

BACTH	Bacterial two hybrid
DTT	Ditiotreitol
EDTA	Ethylenediamine tetraacetic acid
FC	Fold change
IPTG	Isopropyl ß-D-1-thiogalactopyranoside
RNA-Seq	High-throughput RNA sequencing
RT-PCR	Reverse transcription PCR
RT-qPCR	Reverse transcription quantitative PCR
SEC-MALS	Exclusion chromatography combined with multi angle light scattering
X-Gal	5-Bromo-4-Chloro-3-Indolyl β-D-Galactopyranoside
