# Supplementary material for: Searching for Biological Function of the Mysterious PA2504 Protein from Pseudomonas aeruginosa
Source: Int J Mol Sci. 2021 Sep 11;22(18):9833. doi: 10.3390/ijms22189833 (PMC8466066; doi:10.3390/ijms22189833)
Supplement: Supplementary file 1 [file ijms-22-09833-s001.zip › supporting information.v6.pdf]

Supporting Materials and Methods

Bacterial transformation

Competent *E. coli* cells were prepared by the standard $CaCl_2$ method and plasmid DNA was introduced by heat shock method [59].

DNA manipulations

Plasmids and *Pseudomonas* genomic DNA were purified using Plasmid Mini Kit and Genomic Mini Kit, respectively (A&A Biotechnology), according to the manufacturer's instructions.

To purify DNA from agarose, gel slices were cut out and processed using the Gel-Out system (A&A Biotechnology) according to the manufacturer's instructions. PCR amplification was performed using high fidelity Pfu DNA polymerase (Promega). Usually 30 cycles of amplification were performed, at reaction conditions depending on the primers and the size of the final amplification product. The PCR products were identified by agarose gel electrophoresis. All primers are listed in Table S2.

SDS-PAGE gel electrophoresis

Routinely, proteins were separated in denaturing conditions by SDS-PAGE method [59] in 12% polyacrylamide gel. For visualization, proteins were stainedwith Coomassie Brilliant Blue.

Western blot

The membrane was blocked for 1 h at room temperature in the TBS (10 mM Tris-Cl pH 8.0, 150 mM NaCl) blocking buffer (TBS, with 7% non-fat milk) and then for 1 h in the blocking buffer containing mouse primary antibody (anti His_6 diluted 1:3000, Sigma). In order to remove unbound antibodies, the membrane was washed four times with TBS containing 0.1% Tween-20 (TBST) and subsequently incubated with secondary anti-mouse antibodies conjugated with alkaline phosphatase (goat anti-mouse for anti-His_6 diluted 1:3000 in TBST). After 1 h, a series of 10-min washes was carried out with TBS, and the membrane was incubated for 10 min in AP buffer (100 mM NaCl, 100 mM Tris-Cl, 5 mM $MgCl_2$ pH 9.5). Proteins were visualized by immersing the membrane in AP buffer containing alkaline phosphatase substrate NBT (nitro blue tetrazolium) and BCIP (5-bromo-4-chloro-3-indolyl-phosphate) substrates (Promega).

Biofilm production assay

Overnight cultures of *P. aeruginosa* PAO1161 and mutant strains were diluted 1:100 in fresh L-broth medium in three replicates, and 100 µl of each diluted culture was transferred into at least 8 wells on a 96-well plate. The plates were incubated statically at 37°C for approximately 20 h. OD_{600} was measured with a plate reader. The medium with planktonic bacteria was removed, the wells were washed three times with PBS, 200 µl of 0.1% crystal violet solution was added to each well and incubated for 30 min at room temperature. The solution was removed, and the wells were washed three times with water and once with PBS. The plates were dried and 150 µl of 96% ethanol was

added to dissolve the bound stain. After 10 min of incubation at room temperature, the solution was mixed by pipetting, OD_{590} was determined, and the OD_{590}/OD_{600} ratio was calculated.

Motility assays

The assays were performed according to [60]. Swimming plates (1% tryptone, 0.5% NaCl, 0.3% agar), swarming plates (0.8% nutrient broth, 0.5% dextrose, 0.5% agar), and twitching plates (1% bactotryptone, 0.5% NaCl, 1.5% agar) were inoculated from fresh overnight cultures on L-agar plates with a sterile toothpick and observed after incubation at 37°C for 24 h. Motility tests were repeated at least three times.

Antibiotic sensitivity tests

Overnight cultures of *P. aeruginosa* PAO1161 and mutant strains were diluted 1:100 in 15 ml of fresh L-broth medium and incubated at 37°C to OD_{600}=0.1. Bacteria were spread evenly on Mueller–Hinton plates (Biomaxima S.A; 17.6 g L^{-1} casein hydrolase, 2.0 g L^{-1} beef extract, 1.5 g L^{-1} starch, 17.0 g L^{-1} agar) to give a homogenous lawn, and antibiotic disks (Oxoid) were placed in the center of each plate. Antibiotics from different groups were used: cephalosporins (ceftazidime 10 µg, (CAZ)); quinolones (ciprofloxacin 5 µg, (CIP)); β-lactams: (imipenem 10 µg, (IPM);meropenem 10 µg, (MEM);piperacillin 100 µg (PRL)); polymyxins (polymyxin B 300 µg) (PB); colistin 10 µg (CT))and aminoglycosides (tobramycin 10 µg, (TOB)). The plates were incubated at 37°C for 20 h, and the diameter of growth inhibition was measured. The tests were repeated several times.

Biolog Phenotype microarrays

The Biolog system allows microarray based differentiation of growth phenotypes. It uses a redox technology, with cell respiration (NADH production) as a reporter. If the cells are metabolically active, they resiprate leading to the reduction of a tetrazolium dye present in the medium and thereby leading to coloration, proportional to the activity. A PM4A microarray was used to determine, in accordance with the manufacturer's instructions, the ability of *P. aeruginosa* to utilize different sulphur sources. (Biolog, Inc., Hayward, CA.). The IF-0 inoculation fluid was used with Dye Mix A, supplemented with sodium citrate (20mM) as a carbon source and leucine (152 mM). If needed, 200 µg ml^{-1} chloramphenicol was added. The cells were inoculated from fresh overnight L-broth plates supplemented with antibiotics if needed. The bacteria were suspended in 15 ml of IF-0 to the transmittance (T) of 42% on a Biolog turbidimeter, and then diluted again six fold by combining with 75 ml of IF-0 to obtain a final density of 85% transmittance. The PM4A microarraywas were inoculated with cell suspensions at 100 µl per well and incubated at 37°C in an OmniLog PM instrument and monitored for colour change in the wells. The *P. aeruginosa ΔPA2504* strain was compared with the wild-type PAO1161strain and the PA2504 overproducing strain with the PAO1161 strain carrying pKGB empty vector.

Table S1. Bacterial strains used in this study

Strain	Description	Reference
	***Escherichia coli* strains**	
S17-1	*recA pro hsdR hsdM* Tp^R Sm^R ΩRPT-Tc::Mu-Km::TnT	[61]
XL1-Blue MRF'	Δ*(mcrA)*183 Δ(*mcrCB-hsdSMR-mrr*)*173 endA1 supE44 thi-1 recA1 gyrA96 relA1 lac* [F' *proAB*	Stratagene
BL21-DE3	*fhuA2 [lon] ompT gal* (λDE3) [dcm] *ΔhsdS* λDE3 =λsBamHIo ΔEcoRI-B *int::(lacI::PlacUV5*::T7 gene 1) i21 *Δnin5*	NEB
	***Pseudomonas aeruginosa* strains**	
PAO1161 RifR	leu-, r-, Rif^R	[62]
ΔrppH	leu-, r-, Rif^R ΔrppH	[5]
ΔPA2504	leu-, r-, Rif^R ΔPA2504	This work
ΔPA2504ΔrppH	leu-, r-, Rif^R ΔPA2504 ΔrppH	This work

Table S2. Primers used in this study

Primer	Sequence	Purpose
Δ25F1	CGGAATTCCTGCAAGTCCTCTGAAG	pAKE2504 construction
Δ25R1	AACTGCAGCGAGGCCATGTCGACT	pAKE2504 construction
Δ25F2	AACTGCAGTGAGCCTCAGCCTG	pAKE2504 construction
Δ25R2	CTGGATCCGAATACGGTGCTCCT	pAKE2504 construction
QE25F	ATGGATCCGCCTCGTGGACCTT	pQE2504 construction
QE25R	GCAAGCTTTCAACCCTCCGGTC	pQE2504 construction
GB25F	CCGGAATTCATGAGAGGATCGCATCACCATC	pKGB2504 construction
GB25R	GCAAGCTTTCAACCCTCCGGTC	pKGB2504 construction
LgfF	GCGAATTCATGCGTAAAGGC	pKGBgfp2504 construction
LgfR	GCAAGCTTTTTGTACAGTTC	pKGBgfp2504 construction
L25F	GCTCTAGAGCCTCGTGGACC	pKGBgfp2504 construction
L25R	GTGAGCTCTCAACCCTCCGG	pKGBgfp2504 construction
KT25F	CGCGGATCCCGCCTCGTGGACCTTG	pKT2504 and p18C2504 construction
KT25R	CCGGAATTCGATCAACCCTCCGGTCCCTG	pKT2504 and p18C2504 construction
NTrpF	CGCGGATCCCGTGATCGATTCCGATGG	pNTrppH construction
NTrpR	CCGGAATTCGAGTCCCGCGCCAGAAG	pNTrppH construction

Table S3 Proteins identified by mass spectrometry analysis from bands cut from SDS-PAGE gel (Figure 8). For band 1 proteins with mass 35-48 kDa are included and for band 2 with mass 17-23.7 kDa. Given are: the Pseudomonas Genome Database ID; protein name; mascot score - reflects the combined scores of all observed mass spectra that can be matched to amino acid sequences within that protein, a higher score indicates a more confident match; queries matched - number of MS/MS spectra that were matched to given protein; unique sequences - number of unique sequences matched; emPAI - relative quantitation of the proteins in a mixture based on protein coverage by the peptide matches in a database search result. Listed are proteins with p-value ≤0.05. Only proteins with two or more queries matched are included.

PDB ID	Name	Mass (kDa)	Score	Queries Matched	Unique sequences	emPAI
			Band 1			
PA4265	tufA	43.4	18091	336	28	42.26
PA4407	ftsZ	41.2	798	17	12	3.64
PA2612	serS	47.2	758	16	11	2.19
PA0266	davT	45.2	611	12	9	1.53
PA4673	PA4673	40.1	552	13	10	1.85
PA4238	rpoA	36.6	524	12	10	2.53
PA3977	hemL	45.4	468	10	7	1.53
PA0425	mexA	41.0	427	7	7	1.06
PA4583	PA4583	44.4	367	8	8	1.13
PA3364	amiC	42.8	351	8	7	0.99
PA0546	metK	42.7	337	7	6	0.80
PA2127	cgrA	47.6	327	7	7	0.85
PA2623	icd	45.6	324	9	9	1.30
PA2950	fabV	43.5	310	6	6	0.79
PA2760	oprQ	46.9	289	6	5	0.57
PA2195	hcnC	45.3	284	6	5	0.59
PA4942	hflK	44.0	282	5	5	0.62
PA4785	PA4785	45.9	267	7	7	0.90
PA3617	recA	36.9	257	6	6	0.98
PA0302	spuF	42.8	257	5	4	0.63
PA2239	pslI	40.6	229	6	6	0.86
PA4408	ftsA	44.6	205	4	4	0.46
PA5304	dadA	47.1	184	5	5	0.56
PA1373	fabF2	43.5	181	4	4	0.47
PA4677	PA4677	46.9	179	5	5	0.56
PA5258	PA5258	40.8	171	5	5	0.68
PA3478	rhlB	47.1	149	4	4	0.43
PA2664	fhp	43.7	147	4	4	0.47
PA2965	fabF1	43.5	145	3	3	0.34
PA1609	fabB	42.8	139	4	3	0.48
PA0971	tolA	37.9	129	2	2	0.25

PA2945	PA2946	39.5	127	4	4	0.50
PA3891	opuCA	43.3	126	2	2	0.21
PA0354	PA0354	43.8	119	4	4	0.47
PA4939	PA4939	42.5	115	3	3	0.35
PA4498	mdpA	44.1	98	2	2	0.21
PA4786	PA4786	47.2	97	3	3	0.31
PA3147	wbpJ	45.3	95	3	3	0.32
PA1580	gltA	47.7	95	3	3	0.30
PA4302	tadA	46.8	87	2	2	0.20
PA3814	iscS	44.7	81	3	3	0.33
PA3949	PA3949	42.4	80	2	2	0.22
PA2345	PA2345	44.7	66	2	2	0.21
PA3981	PA3981	38.4	64	2	2	0.24
PA1288	PA1288	45.6	60	2	2	0.20
**PDB ID**	**Name**	**Mass (kDa)**	**Score**	**Queries Matched**	**Unique sequences**	**emPAI**
			Band 2			
PA4239	rpsD	23.3	1961	41	17	23.95
PA0139	ahpC	20.5	1277	21	9	5.92
PA4257	rpsC	18.7	957	20	10	4.62
PA1902	phzD2	23.2	913	19	8	5.75
PA2586	gacA	23.6	800	17	9	10.32
PA2000	dhcB	23.2	575	9	3	1.60
PA3152	hisH2	22.7	495	14	5	2.23
PA2126	cgrC	23.7	422	9	5	1.55
PA4440	PA4440	22.3	408	9	4	1.68
PA4256	rplP	20.7	388	7	4	2.13
PA1432	lasI	22.7	312	10	5	2.90
PA2007	maiA	23.7	297	10	8	3.48
PA1683	PA1683	22.9	266	5	3	0.79
PA4428	sspA	23.6	235	5	3	0.75
PA2222	PA2222	23.4	168	3	2	0.46
PA0762	algU	22.2	162	5	3	0.82
PA3624	pcm	23.4	160	5	3	0.77
PA3988	lptE	22.9	158	4	3	0.79
PA4263	rplC	22.6	157	4	3	0.80
PA4762	grpE	20.7	151	4	2	0.54
PA3754	PA3754	22.9	130	3	2	0.48
PA1847	nfuA	21.1	125	3	2	0.52
PA5143	hisB	21.9	97	3	2	0.83
PA3604	erdR	23.5	95	3	3	0.76
PA4449	hisG	22.8	86	2	2	0.48
PA4951	orn	20.8	84	2	1	0.24
PA4493	roxR	20.6	81	2	1	0.24
PA5564	gidB	23.7	77	2	1	0.21
PA1878	PA1878	21.9	73	2	2	0.50

PA4431	PA4432	20.8	70	2	1	0,23
PA3007	lexA	22.5	66	2	2	0,48
PA4047	ribA	22.1	59	2	2	0,49

Supporting Figures

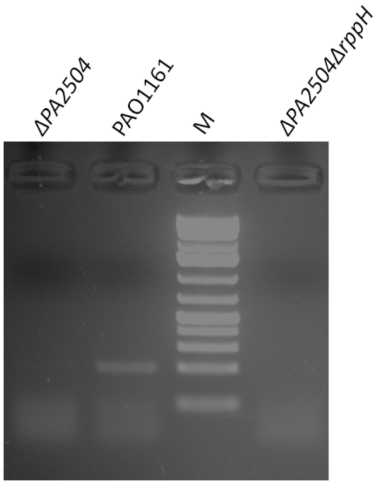

Figure S1. Expression of *PA2504* gene in *P. aeruginosa* wild-type PAO1161 and mutant *ΔPA2504* and *ΔPA2504ΔrppH* strains analyzed by RT-PCR. Wild type PAO1161 cDNA was used as a positive control.

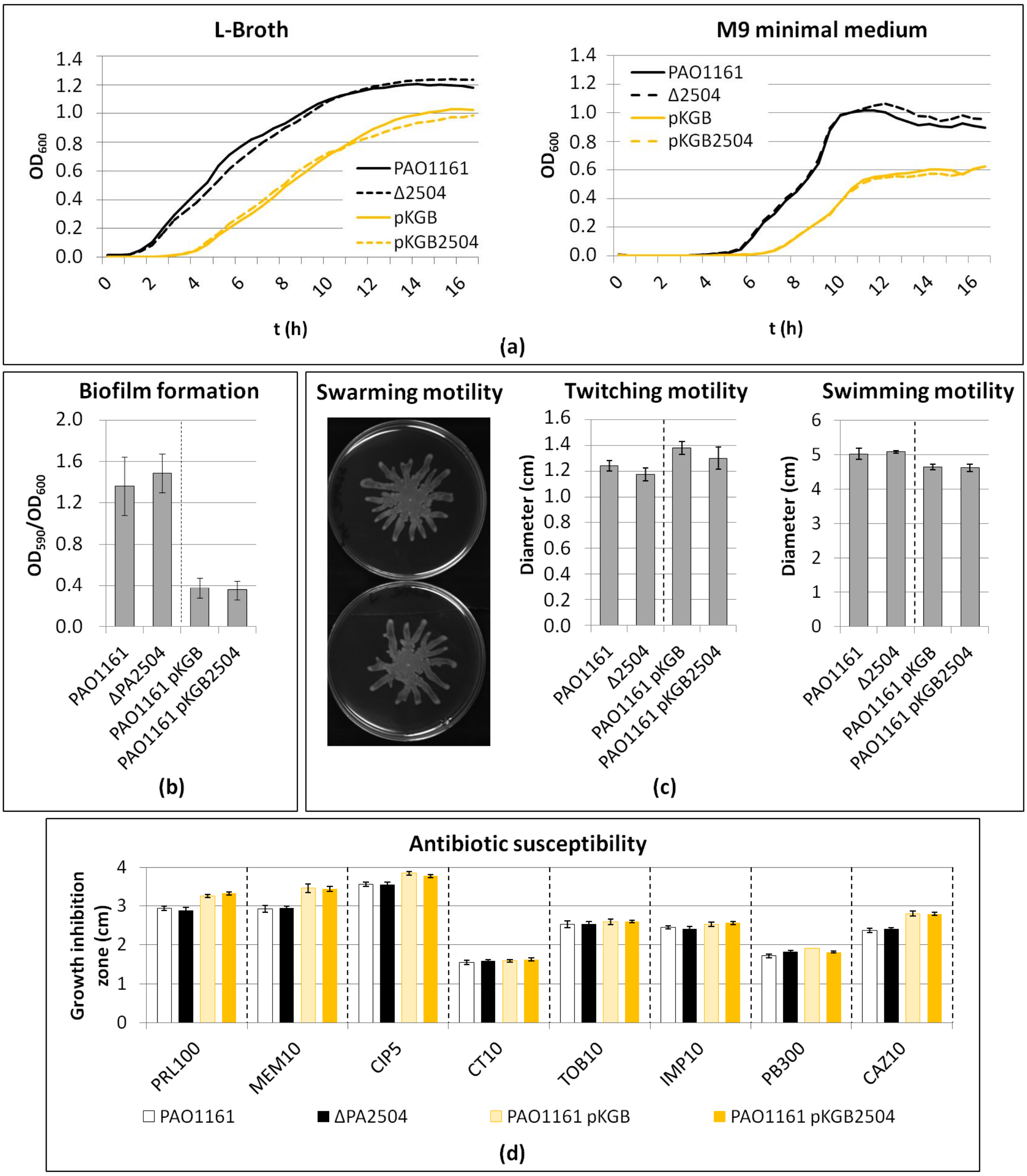

Figure S2. Effect of PA2504 deprivation and overproduction on growth, biofilm production, motility and antibiotic susceptibility of *P. aeruginosa*. **(a)** Growth curves of *P. aeruginosa* strains in L-broth rich medium and minimal M9 medium. **(b)** Effect of PA2504 deprivation and overproduction on biofilm formation capability **(c)** Effect of PA2504 deprivation and overproduction on swimming, swarming and twitching motility **(d)** Effect of PA2504 depravation and overproduction on antibiotic susceptibility. In all experiments wild-type *P. aeruginosa* PAO1161 and *ΔPA2504* mutant were compared as well as the PAO1161 pKGB empty plasmid strain with the PA2504 protein overproducing PAO1161 pKGB2504 strain. All experiments were performed as described in supplementary Materials and Methods.

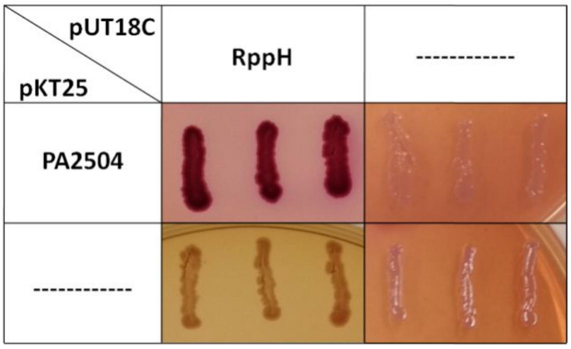

Figure S3. Interaction between PA2504 and RppH visualized with the use of BACTH system on MacConkey selective medium (red colonies)**.** Negative control **-** expression of PA2504 or RppH in the presence of empty pUT18C and pKT25 vectors and co-transformation with two empty pKT25 and pUT18C vectors.

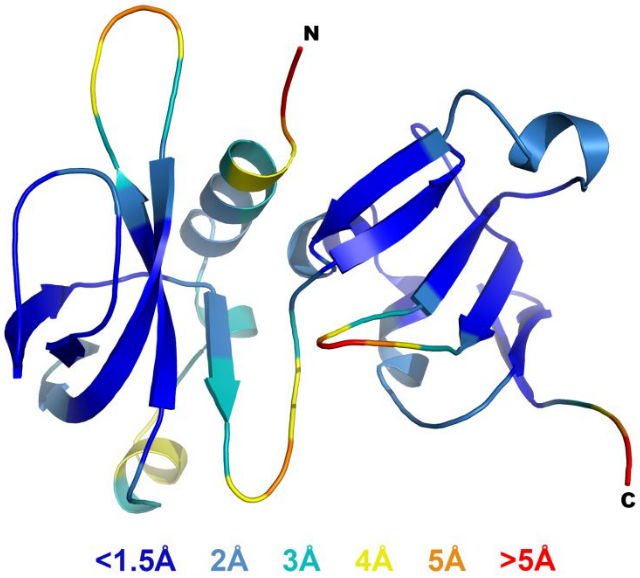

Figure S4. 3D model of PA2504 protein coloured according to trRosetta error estimates. The values reflect prediction uncertainties and in the case of homology modelling provide a distance between the model and the template.

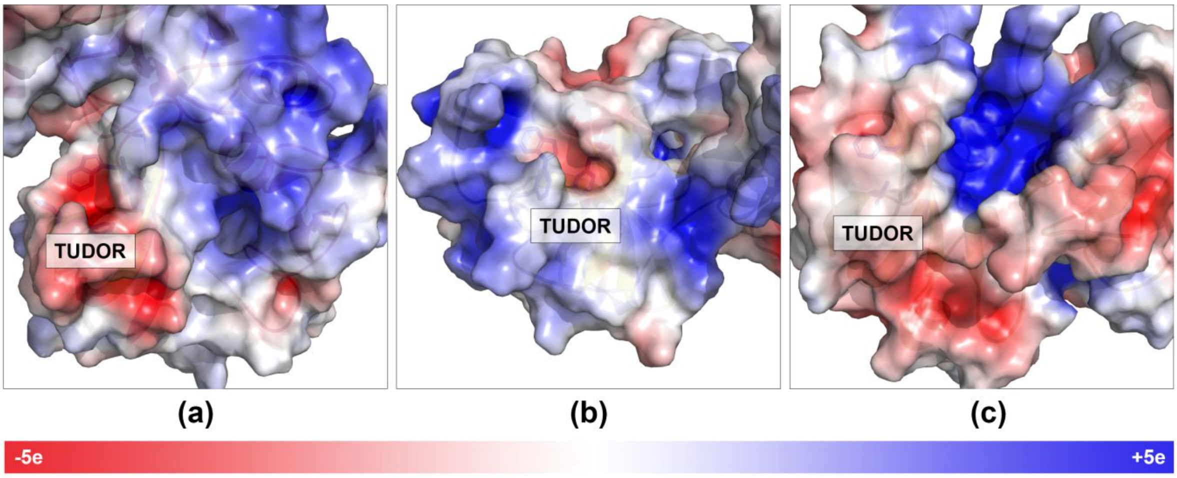

Figure S5. Molecular surfaces of TUDOR domains of (A) PHF1, (B) ZMYND8 and (C) PA2504 coloured according to calculated electrostatic potential. Molecules were rotated to ensure same orientation of TUDOR domain.

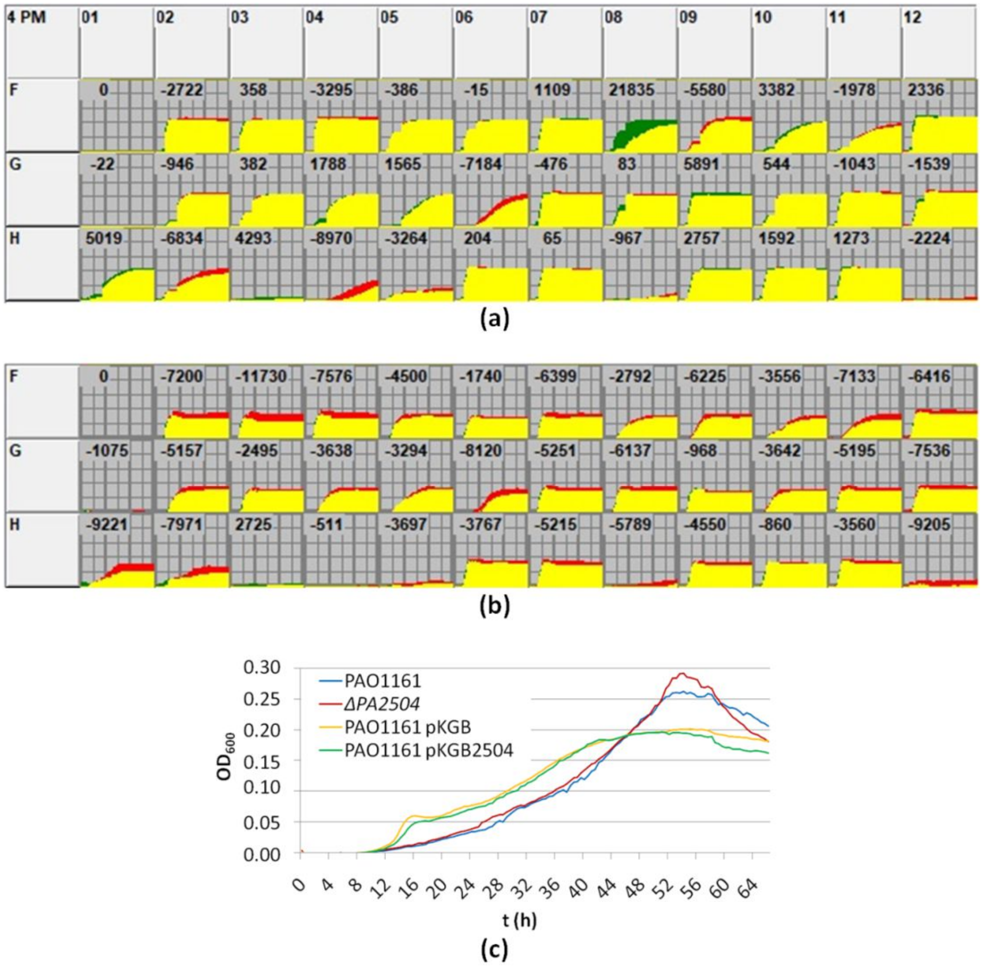

Figure S6 Effect of PA2504 deprivation and overproduction on *P. aeruginosa* growth on different sulphur sources. PA4A Biolog system phenotype microarray was used to determine metabolic activity. (**a**) Comparison of ΔPA2504 mutant(red)withwild type PAO1161 (green) strain. Yellow marks overlapping parts of curves. Difference between the strains is in well F8 with D-cysteine. **(b)** Comparison of the *P. aeruginosa* PAO1161 carrying pKGB empty plasmid (green) with the PAO1161 carrying pKGB2504 strainoverexpressing PA2504 (red). Yellow marks overlapping parts of curves. The sulphur sources present in each well were: **F1-** none, negative control; **F2-** sulphate; **F3** – thiosulphate; **F4** – tetrathionate; **F5** – thiophosphate; **F6** - dithiophosphate; **F7** - L-cysteine; **F8** - D-cysteine; **F9** - L-cysteinyl-glycine; **F10** - L-cysteic acid; **F11** – cysteamine; **F12** - L-cysteine sulphinic acid; **G1-** *N*-acetyl-L-cysteine; **G2** - *S*-methyl-L-cysteine; **G3** – cystathionine; **G4** – lanthionine; **G5** – glutathione; **G6** - D,L-ethionine; **G7** - L-methionine; **G8** - D-methionine; **G9** - glycyl-L-methionine; **G10** - *N*-acetyl-D,L-methionine; **G11** - L-methionine sulphoxide; **G12** - L-methionine sulphone; **H1** – L-djenkolic acid; **H2** – thiourea; **H3** - L-thio-ß-D-glucose; **H4** d,l-lipoamide; **H5** - taurocholic acid; **H6** - taurine; **H7** – hypotaurine; **H8** - *P*-amino benzene sulphonic acid; **H9** - butane sulphonic acid; **H10** - 2-hydroxyethane sulphonic acid; **H11** - methane sulphonic acid; **H12** – tetramethylene sulphone. **(c)** Growth curves of the *P. aeruginosa* strains in modified M9 minimal medium supplemented with 0.5 mM D-cysteine. The experiments were performed as described in supplementary Materials and Methods.

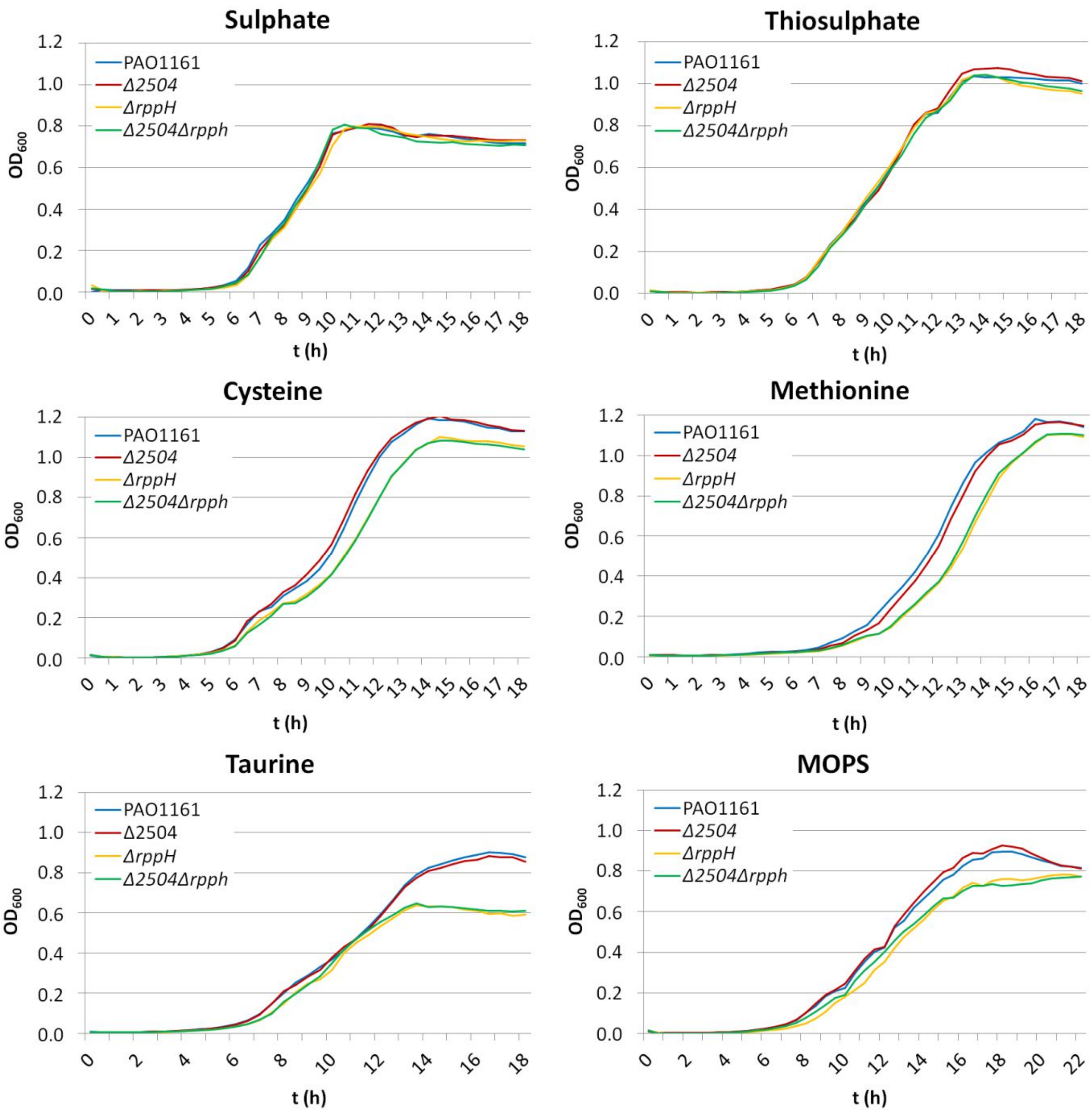

Figure S7. Growth curves of the *P. aeruginosa* wild-type PAO1161 and *ΔrppH*, *ΔPA2504* and *ΔPA2504ΔrppH* mutant strains in modified M9 minimal medium supplemented with sulphur source indicated (0.5 mM).

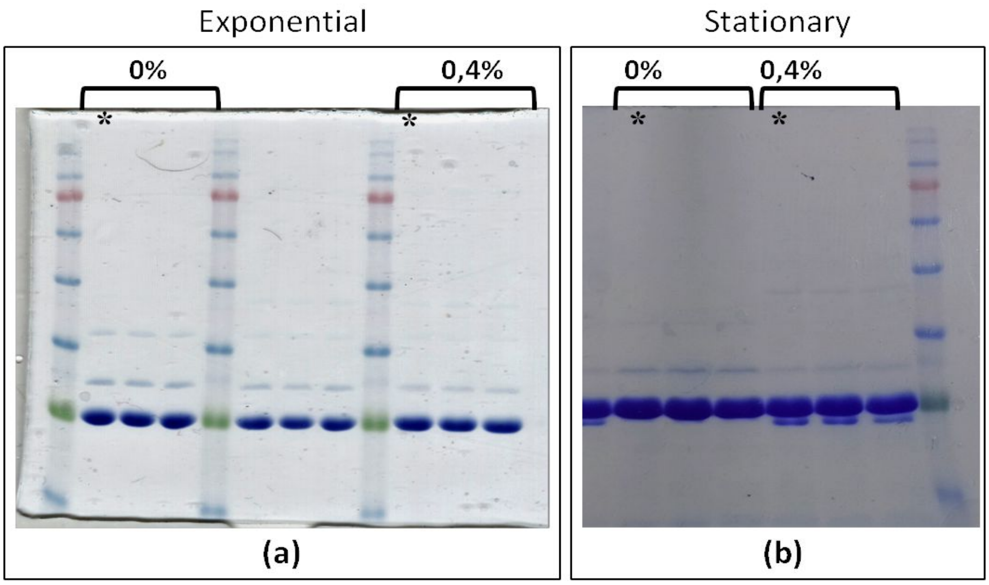

Figure S8. Uncropped pictures of SDS-PAGE gels used to prepare main text figure 8. (a) exponential phase of growth. (b) stationary phase of growth. Lanes used to prepare Figure 8 are marked with asterisk.

Supporting reference list

59. Sambrook, J.; Fritsch, E.F.; Maniatis, T. *Molecular Cloning: A Laboratory Manual* 2nd. ed.; Cold Spring Harbor Laboratory Press: Cold Spring Harbor, NY, USA, 1989; ISBN 978-0-87969-309-1.
60. Rashid, M.H.; Kornberg, A. Inorganic Polyphosphate Is Needed for Swimming, Swarming, and Twitching Motilities of Pseudomonas Aeruginosa. *Proc. Natl. Acad. Sci. USA* **2000**, *97*, 4885–4890, doi:10.1073/pnas.060030097.
61. Simon, R.; O'Connell, M.; Labes, M.; Pühler, A. Plasmid vectors for the genetic analysis and manipulation of rhizobia and other gram-negative bacteria. In *Methods in Enzymology*; Elsevier: Amsterdam, The Netherlands, 1986; Volume 118, pp. 640–659; ISBN 978-0-12-182018-3.
62. Lasocki, K.; Bartosik, A.A.; Mierzejewska, J.; Thomas, C.M.; Jagura-Burdzy, G. Deletion of the *ParA* (*Soj*) Homologue in *Pseudomonas Aeruginosa* Causes ParB Instability and Affects Growth Rate, Chromosome Segregation, and Motility. *J. Bacteriol.***2007**, *189*, 5762–5772, doi:10.1128/JB.00371-07.
